# Orthokeratinized odontogenic cysts: a Spanish tertiary care centre study based on HPV DNA detection

**DOI:** 10.1186/s13005-018-0167-3

**Published:** 2018-07-13

**Authors:** Beatriz Vera-Sirera, Luis Rubio-Martínez, Leopoldo Forner-Navarro, Francisco Vera-Sempere

**Affiliations:** 1 Department of Stomatology, Faculty of Medicine and Dentistry, University of Valencia, Valencia, Comunidad Valenciana Spain; 2 Molecular Pathology Unit, Department of Pathology, La Fe University Hospital, University of Valencia, Valencia, Comunidad Valenciana Spain; 3 Department of Pathology, Faculty of Medicine and Dentistry, La Fe University Hospital, University of Valencia, Torre A, 2° planta, Avda. Fernando Abril Martorell 106, 46026 Valencia, Comunidad Valenciana Spain

**Keywords:** Orthokeratinized odontogenic cyst, Recurrence, HPV, High- and low-risk

## Abstract

**Background:**

The role of human papillomavirus (HPV) in orthokeratinized odontogenic cysts (OOCs) has rarely been studied. The objective is to describe the clinicopathological findings in a series of OOCs from a Spanish population that were investigated in relation to the possible presence of HPV.

**Methods:**

A clinicopathological retrospective analysis followed by a molecular analysis of 28 high- and low-risk HPV genotypes was performed in OOC samples of patients seen during the last 15-years in a Spanish tertiary care center.

**Results:**

Of 115 odontogenic cysts with keratinization, 16 cases of OOCs were confirmed and evaluated. OOCs occurred predominantly in the mandible of males (mean age 36.06 ± 13.16 years). Swelling of the jaw followed by pain were the most common clinical symptoms, and 56.5% of the OOC cases were associated with an unerupted tooth. After a mean post-cystectomy follow-up of 3.8 years, only one recurrent case was observed, resulting in a verrucous cystic lesion that was considered premalignant after immunohistological examination. DNA extraction was successful from 14 of the 16 OOC cases. None of the primary OCCs or the single recurrent OOC were positive for HPV in the molecular analysis.

**Conclusions:**

OOCs show a very limited potential for recurrence. Our results suggest that neither high- or low-risk HPV subtypes are likely to play a role in the etiology or neoplastic transformation of OOC, at least in the Spanish population.

## Background

Orthokeratinized odontogenic cyst (OOC) is a rare intraosseous cyst characterized by an orthokeratinized epithelial lining and minimal clinical aggressiveness [1]. OOCs were first described in 1927 by Schultz [2] as a variant of odontogenic keratocysts, now known as keratocystic odontogenic tumours (KCOTs) [3]. It was not until 1981 that Wright [4] described their clinicopathological aspects, indicating that OOCs are a distinct entity from odontogenic keratocysts. Several series of OOCs have been reported, confirming that their distinctive clinical, histopathological and biological features differ substantially from those of odontogenic keratocysts, as well as demonstrating a better prognosis and lower recurrence rates [1, 5, 6].

Nonetheless, rare cases of bilateral [7], multiple [8] and recurrent [9] OOCs have been reported, and although OOCs are considered clinically to be minimally aggressive, both dysplastic and malignant transformation have been reported [10]. OOCs sometimes transform into an uncommon type of well-differentiated squamous carcinoma known as carcinoma cuniculatum [11].

Over the last two decades, keratinizing cysts from various sites have been evaluated to assess a possible role of human papillomavirus (HPV) in their development and malignant transformation [12]. Reports are also available on odontogenic keratocysts [13] as well as carcinoma cuniculatum [14], in which specific HPV subtypes have been detected.

In this study, we evaluated the clinicopathological profile of OOCs seen at our institution over the last 15 years and analysed the possible presence of HPV.

## Methods

This study was approved by our institutional Ethics Committee for Biomedical Research (protocol no. 2013/0045). Files of the Department of Pathology at La Fe University Hospital (Valencia, Spain) from 2000 to 2015 were reviewed. A retrospective search of a pathological diagnosis database (Pat Win® v4.6.0), employing the search terms “keratocyst”, “primordial cyst”, “keratocystic odontogenic tumor”, “orthokeratinized odontogenic cyst”, and “keratinized cyst”, to reflect changes in terminology over time, identified 115 odontogenic cysts with keratinization.

### Histological and clinical revision

Following a histological review of these 115 cases, 16 cases of OOC were selected based on the morphological criteria established by Wright [4]. Lesions in which all or a predominant portion of the epithelial lining showed non-corrugated orthokeratinization, with the presence of a granular layer, were included in this series. The presence of this orthokeratinization on the epithelial surface as well as of a granular layer in the thickness of the epithelium was clearly demonstrated with PAS staining in all selected cases. Likewise, we also included lesions in which basal and parabasal cells were not prominent and did not palisade or polarize. Cystic lesions containing only focal OOC areas or various skin appendages (i.e. hair follicles and sebaceous and sweat glands) were excluded.

Clinical data, including age, sex, lesion location (maxilla or mandible) (anterior, premolar or molar regions), radiologic features, surgical procedures, and information on recurrence (including optical and immunohistochemical data obtained from the recurrent OOC samples), were reviewed.

### Immunohistochemistry

Our immunohistochemical study, performed on recurrent OOC samples included the analysis of two well-known markers (p53 and Ki67), both of which are implicated in tumorigenesis and cell proliferation [6]. Briefly, 5-μm sections were cut from the original paraffin-embedded blocks and mounted on poly-L-lysine-coated glass slides prior to immunohistochemical staining, performed using monoclonal antibodies: mouse antihuman p53 (clone DO-7, dilution 1:50, Dako®, Glostrup, Denmark) and antihuman Ki67 (clone MIB-1, dilution 1:100, Dako®, Glostrup, Denmark). Immunostaining was visualized using the high-pH EnVision FLEX system (Dako®, Glostrup, Denmark); hematoxylin was used for counterstaining and for both techniques tonsil sections with oropharyngeal epithelium were employed as positive staining controls and the negative controls were mock-stained test sections (the primary antibody was replaced with PBS).

### Amplification and detection of HPV DNA

After histological and clinical revision, formalin-fixed, paraffin embedded (FFPE) blocks of all selected OOC cases, including the one recurrent OOC, were retrieved from the archives of the Department of Pathology. The FFPE blocks were cut into three 5-μm sections, and DNA extraction was performed using a MagCore® Super HF16 automated nucleic acid extractor (RBC Bioscience Corp., New Taipei City, Taiwan).

For HPV detection and genotyping, an Anyplex II HPV28 (CE-IVD) kit (Seegene, Seoul, South Korea) was used to identify 14 high-risk and 14 low-risk HPV genotypes in two PCR reactions: mixture A (types 16, 18, 31, 33, 35, 39, 45, 51, 52, 56, 58, 59, 66 and 68) and mixture B (types 6, 11, 26, 40, 42, 43, 44, 53, 54, 61, 69, 70, 73 and 82), respectively. This semi-quantitative multiplex real-time PCR Anyplex II HPV28 system uses both dual priming oligonucleotides and tagging oligonucleotide cleavage and extension technology that combines five dyes and seven different melting-curve temperatures to distinguish the 28 HPV genotypes. PCR reactions were performed on a CFX96 real-time thermocycler (Bio-Rad Laboratories, Hercules, CA, USA) using 5 μL DNA for both mixtures A and B.

As internal control, the L1 gene of HPV DNA was simultaneously co-amplified with the human beta-globin housekeeping gene in the same PCR reaction tube. In addition, two positive controls (high-risk and low-risk HPV genotypes) were included in each PCR run along with two negative controls (non-template control) from the DNA extraction step and PCR experiment. Finally, all data from the PCR runs were interpreted using Seegene viewer software supplied by the manufacturer (Seegene, Seoul, South Korea).

## Results

### Clinicopathological data

**Fig. 1:**
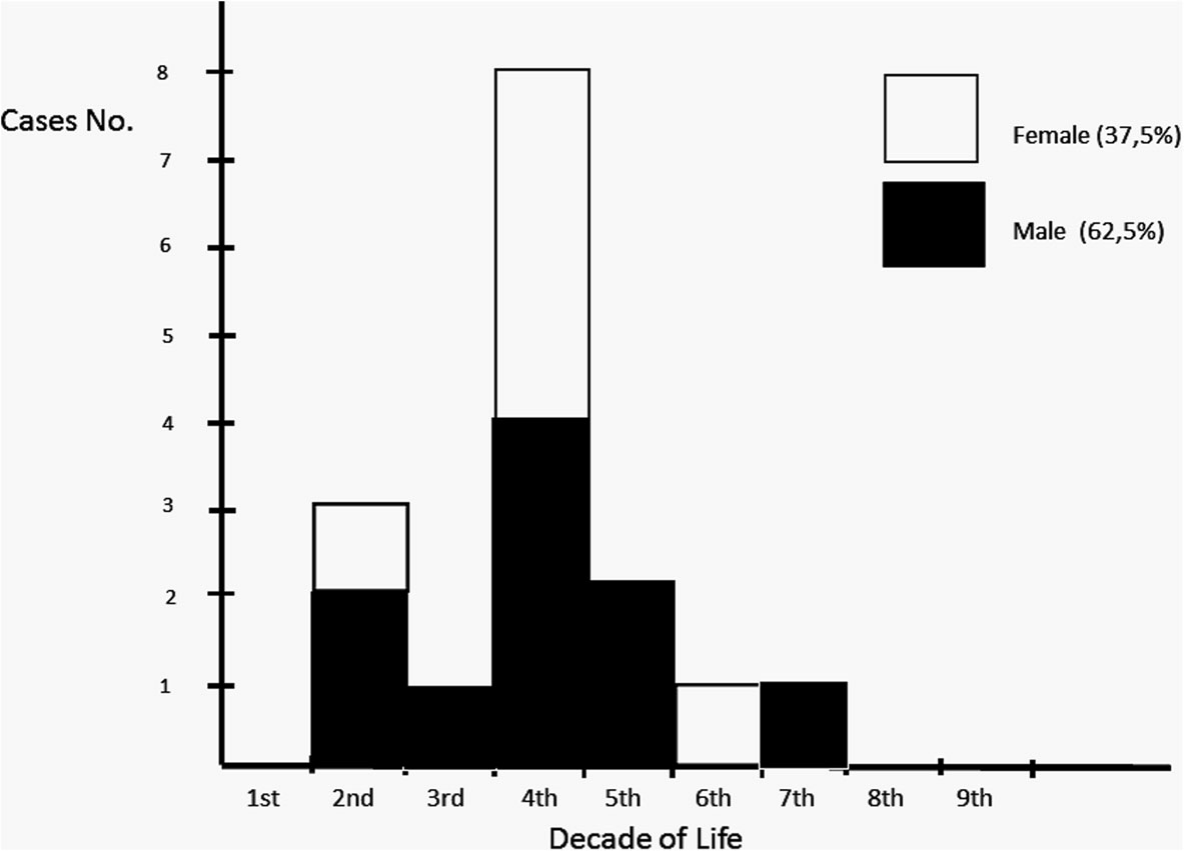
Distribution of OOC cases by sex and age

The histological review of 115 keratinizing jaw cysts detected only 16 OOCs (13.91%). The cases were accepted for inclusion as OOC if they fulfilled the criterion of a complete or predominant epithelial lining with non-corrugated orthokeratinization and the presence of a granular layer. Of these 16 patients, 10 were male and 6 were female (male-to-female ratio, 1.66:1). Age at diagnosis ranged from 13 to 65 years (average 36.06 ± 13.16 years), with a clear predilection for the fourth decade of life (50%) in both sexes (Fig. 1). The mandible was affected in 12 cases (75%) and the maxilla in 4 (25%). Molars (65%) and premolars (16%) were the most commonly affected regions. Only one case showed bilateral involvement of two mandibular cystic lesions, which were diagnosed simultaneously. Of the 16 OOCs, 9 (56.5%) were associated with an impacted tooth, of which 6 were interpreted clinically as dentigerous cysts.

Jaw swelling was the most common symptom (12 cases; 75%). Four (25%) patients also complained of pain. The evolution of the symptoms ranged from 20 days to 5 years (mean, 9 months), with 7 patients (43.75%) having an evolution of less than 3 months. Interestingly, a maxillary OOC manifesting with odontogenic sinusitis was initially considered clinically and radiologically to be a periapical inflammatory cyst.

Enucleation, with or without curettage, was performed in 13 of the OOC cases; 1 case required marsupialization followed by enucleation, and the remaining 2 cases underwent a peripheral ostectomy because of their relatively large size. Related follow-up data were available for all patients. The follow-up period ranged from 12 to 120 months, average 31.12 months, during which only one case (1/16; 6.25%) recurred. We describe this case in detail below.

**Fig. 2:**
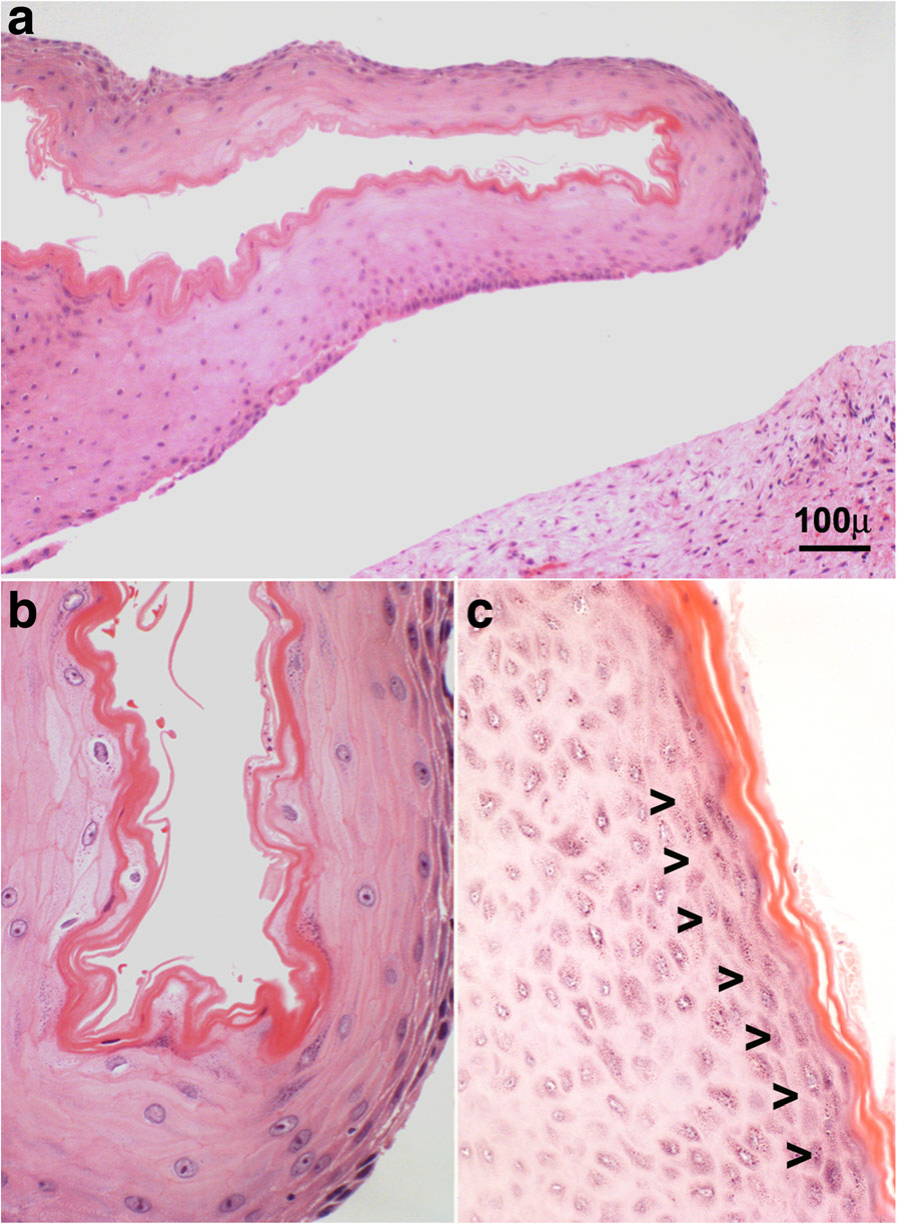
Histological image of a cystic lesion corresponding to an OOC (**a**), showing orthokeratotic keratinization (**b**) with the presence of a granulosa layer (arrows) (**c**) (hematoxylin and eosin, 100× and 400×)

**Fig. 3:**
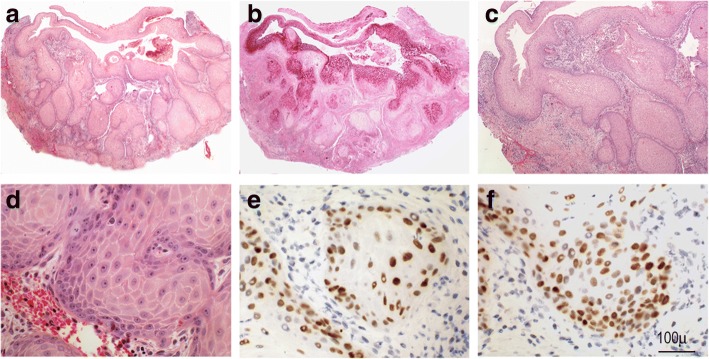
Recurrence of an OOC. Panoramic view showing the wall of a cystic lesion with large areas of hyperplastic verrucous growth with central filling by keratin (**a**), which is highlighted by PAS staining (**b**). Hyperplastic verrucous growth shows an endophytic and “pushing” well-differentiated pattern (**c**). Deeper portions showed epithelial nests with mild atypia and basement membrane integrity (**d**), which in the immunohistochemical analysis demonstrated proliferative cellular activity (Ki67 positivity) not restricted to the basal cell layer (**e**), as well as diffuse nuclear p53 reactivity (**f**)

The only recurrence in our series was a teenage male (13 years old) who presented with a left mandibular radiolucent lesion measuring 30 mm, which displaced the roots of neighbouring molars without paresthesia, pain, or tooth mobility. Initially, an intra-oral double exodontia with cystectomy was performed. Histological examination revealed an odontogenic cyst lined by a stratified epithelium showing orthokeratotic keratinization on its surface as well as the presence of an evident layer of granular cells (Fig. 2a-c). Eight months after the cystectomy, the patient reported a painful swelling at the site of the operation. Panoramic radiography showed an increased radiolucent mandibular area with a poorly defined lower edge; new and wider curettage of the lesion was performed. Histological examination of the recurrent lesion revealed an OOC with features of verrucous hyperplasia. Distinct endophytic and “pushing” invasion together with a well-differentiated pattern of verrucous appearance showing dysplastic features with mild cellular atypia was observed, although there was no rupture of the basement membrane. Proliferative cellular activity (positive for Ki67) was not restricted to basal cells, and p53 expression was observed in all cell nuclei in the immunohistochemical analysis (Fig. 3a-f). These morphological changes were interpreted as early signs of malignization, although there was no evidence of invasive growth based on basement membrane integrity. After surgery, clinical and radiological long-term follow-up of the patient was planned. After 2 years of quarterly visits, there was no evidence of growth or new recurrence.

### Amplification and detection of HPV DNA

In total, 38 FFPE blocks from the 16 OOC cases (including the recurrent case 13) were used for DNA extraction. Two of the 16 OOCs (cases 5 and 7) were invalid for the Anyplex II HPV28 test due to lack of beta-globin amplification and were excluded from the study (see Table 1). For the remaining 14 cases, we performed HPV analysis in 32 FFPE blocks with good DNA quality, but observed no HPV positivity in any of the primary OOCs or the recurrent lesion.

**Table 1:** Clinicopathological characteristics and HPV genotyping results

Case	Age	Gender	Location	Primary/Recurrence	# FFPE	year	HPV 28	IC^a^
1	38	Female	Mandible	P	1	2007	negative	1
2	34	Female	Maxilla	P	2	2002	negative	1
3	39	Male	Mandible	P	3	2004	negative	3
4	37	Female	Mandible	P	1	2006	negative	1
5	18	Female	Mandible	P	1	2007	ND	0
6	42	Male	Mandible	P	2	2006	negative	2
7	25	Male	Mandible	P	1	2007	ND	0
8	36	Female	Mandible	P	1	2011	negative	1
9	38	Male	Mandible	P	2	2006	negative	2
10	19	Male	Mandible	P	6	2001	negative	5
11	35	Male	Mandible	P	2	2014	negative	2
12	45	Male	Mandible	P	2	2014	negative	2
13	14	Male	Mandible	P&R	4 & 5	2015 / 2016	negative	4 & 5
14	39	Male	Mandible	P	1	2016	negative	1
14	45	Male	Mandible	P	3	2014	negative	2
16	65	Male	Maxilla	P	1	2013	negative	1

IC^a^: DNA Internal Control (number of the FFPE bloks showing a positive beta-globin gene amplification)

## Discussion

This study, complemented by molecular detection of HPV DNA, examined the largest series of OOCs affecting a Spanish population reported to date.

Of our odontogenic cysts with keratinization, 13.91% were identified as OOCs. This incidence agrees with those previously reported for OOCs (range 5.2–16.8%) among cases previously coded as KCOT [1, 5].

The average age at diagnosis of our patients was 36.06 ± 13.16 years with a tendency to occur during the fourth decade (50%). There was a male predominance, which is consistent with most published series [1, 4, 5, 9]. More than half our OOC cases were radiographically associated with an impacted tooth, a characteristic observed at varying rates in previous reports, with an average incidence of approximately 60.8% [1]. Of note in our series, a maxillary OOC that manifested clinically as odontogenic sinusitis was radiologically considered a periapical inflammatory cyst. Thus indicating that OOC should be included in the differential diagnosis of radiolucencies in the periapical region, especially considering that up to seven cases of an OOC resembling a radicular cyst have been described previously [15].

Regarding the evolution of OOCs, our study confirmed the low rate of recurrence of these cysts, with only one recurrence observed among 16 patients who were followed up after surgery for 12–120 months (average 31.12 months). This low recurrence rate is somewhat higher than the reported mean of 4%, based on pooled data from OOC series with adequate follow-up [9]; however, it is less than the overall rate reported for KCOTs (12–60%) [1]. This difference in the recurrence rate is important, since the morphological distinction of OOCs from KCOTs is supported mainly by studies that indicated a significantly lower rate of OOC recurrence following surgery [1, 4, 5].

Although rare, recurrence of OOC is possible, and may be indicative of malignant transformation. In our series, there was one recurrence observing hyperplastic verrucous changes in the lining of the OOC. These findings were histopathologically interpreted as atypical based on the overexpression of p53 as well as by the presence of proliferative activity in suprabasal cells. This case is unusual considering the young age of the patient, existence of a prior OOC, and the short period from the original OOC to recurrence; this supports the importance of performing additional microscopic studies of all resected cystic lesions in the jaws and the need for careful follow-up after their removal [10].

Previous studies have reported the appearance of verrucous proliferation in OOCs [16], as well as the possible emergence of squamous cell carcinomas (SCCs) from pre-existing OOCs [17]. Cysts with keratinization have a higher incidence of malignization than other OOCs [18], often presenting as highly differentiated SCCs [17], including cases of verrucous [19] or cuniculatum carcinoma [20], with some well-documented cases arising from OOCs [20–23]. Thus, although OOCs are considered clinically less aggressive lesions than KCOTs, the epithelial lining of OOCs may have neoplastic potential [21].

The pathogenesis of malignant transformation of OOC epithelia remains unclear [17]. Different hypotheses, such as long-standing chronic inflammation [17], genetic mutations in exon 6 of the *TP53* gene [23] or oncogenic viral effects [13] have been suggested as predisposing factors, but the specific underlying mechanisms of this carcinogenesis remain unclear. It is thought that HPV may be associated with the pathogenesis of keratinizing OOC [13] and carcinoma cuniculatum [14], as well as some other carcinomas arising from epidermal cysts [12]. In addition, HPV may play a role in the development of verrucous proliferation in various sites, including oral locations [16, 24, 25]; however, this remains controversial [26].

In agreement with previous reports [16, 24, 27], we did not detect HPV genotypes in our molecular analysis of atypical verrucous hyperplasia in the recurrent case, suggesting that neither high- or low-risk HPV subtypes are likely to play a role in the pathogenesis of atypical verrucous changes that may develop into OOC recurrence. Moreover, molecular analysis did not detect HPV involvement in our series of primary OOC samples with successful DNA extraction. Thus, it is unlikely that HPV plays a role in the pathogenesis of OOCs, at least in the Spanish population. However, we cannot completely exclude that HPV is involved in OOCs in patients from other geographic areas, as commonly occurs in other virus-related disorders [28]. Additional epidemiological and molecular investigations in a large series of patients with OOCs of different ethnicities using molecular analysis of a broad spectrum of HPV types are required to confirm our results.

## Conclusions

OOCs are a rare type of odontogenic cyst that show minimal clinical aggressiveness. Nonetheless, although very rare, recurrence is possible and can be a source of malignant transformation. Neither high- or low-risk HPV subtypes are likely to play a role, at least in the Spanish population, in the etiology of an OOC or in its neoplastic transformation.

## Abbreviations

FFPE: Formalin-fixed, paraffin embedded
HPV: Human papillomavirus
KCOT: Keratocystic odontogenic tumour
OOC: Orthokeratinized odontogenic cyst
PCR: Polymerase chain reaction
SCC: Squamous cell carcinomas
